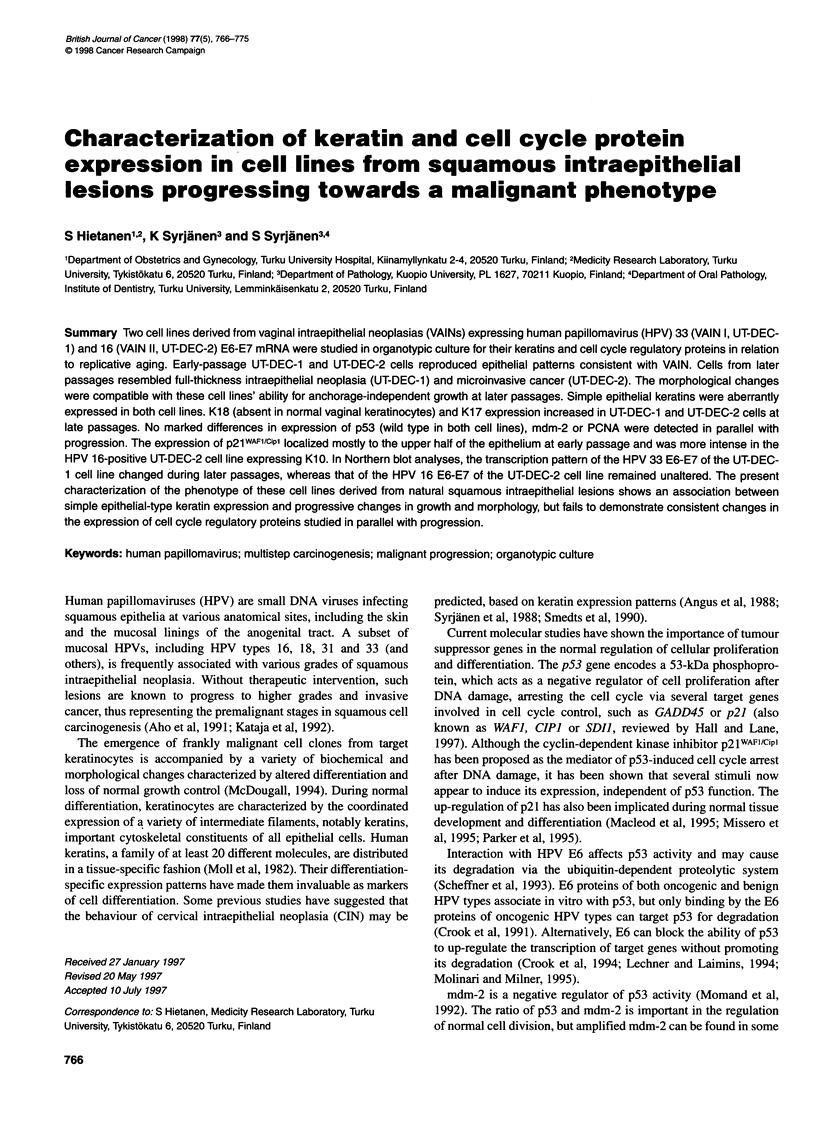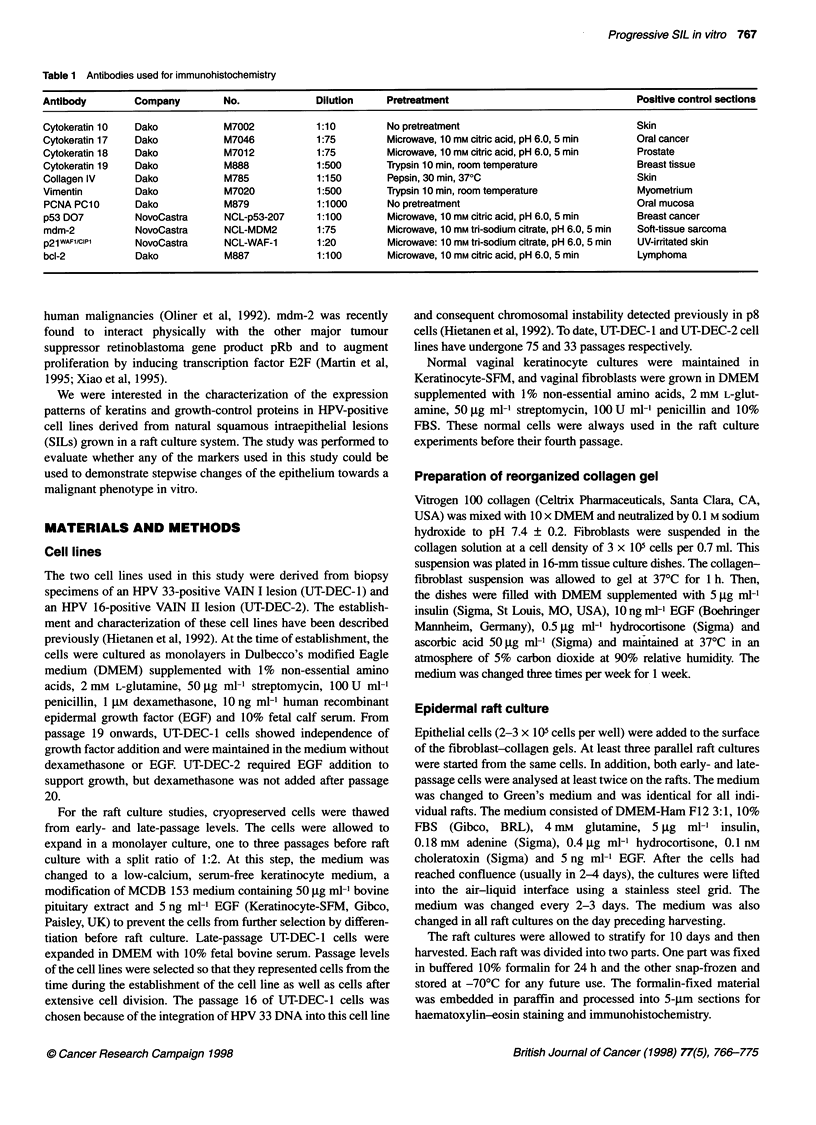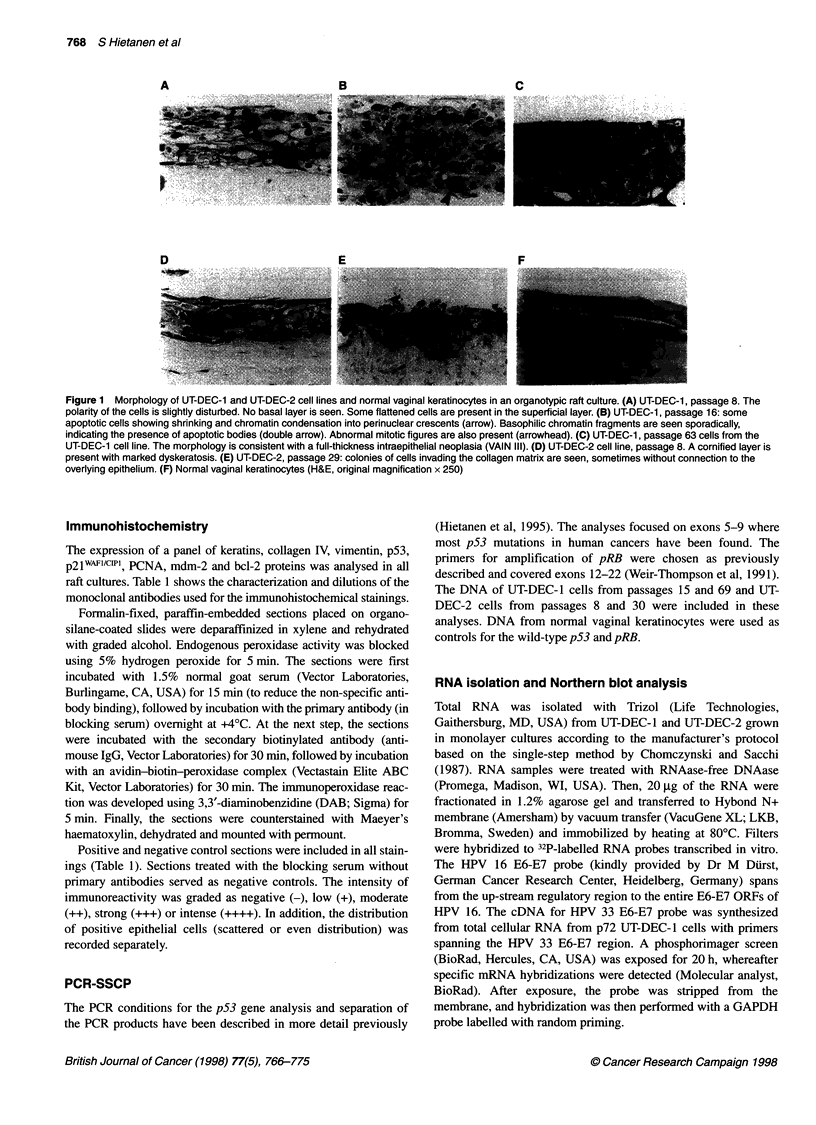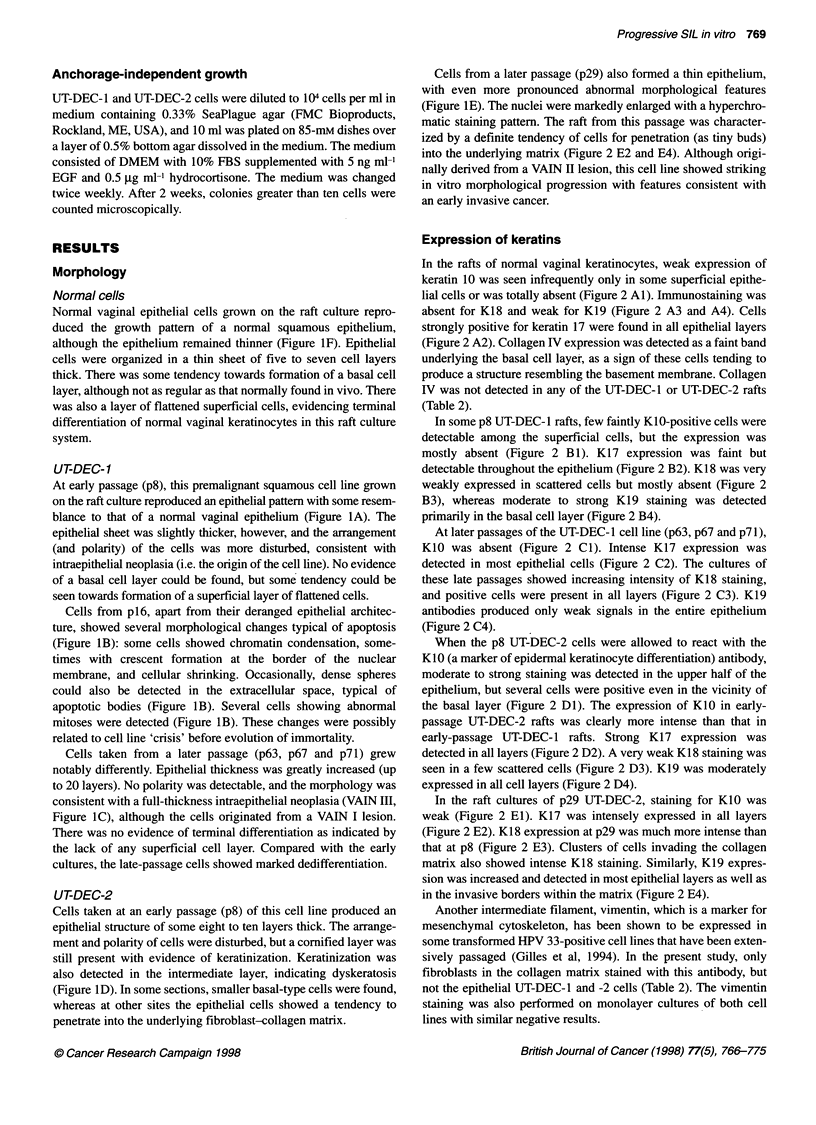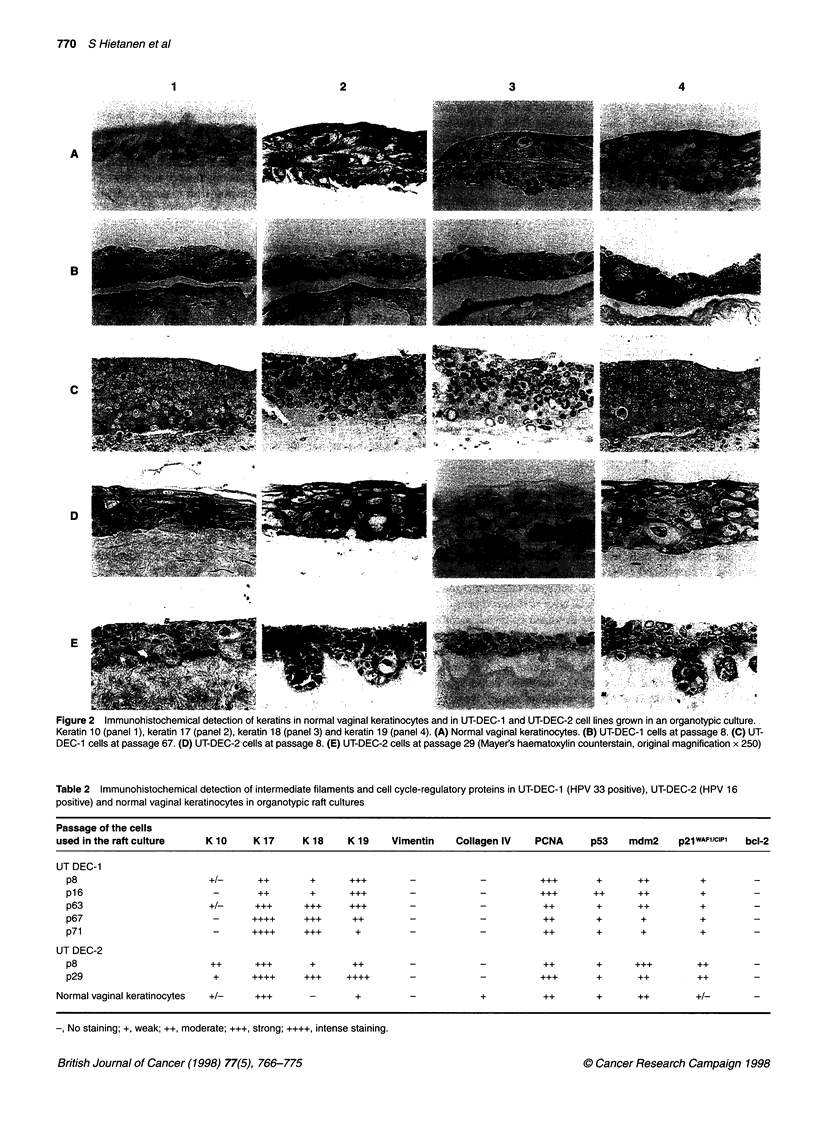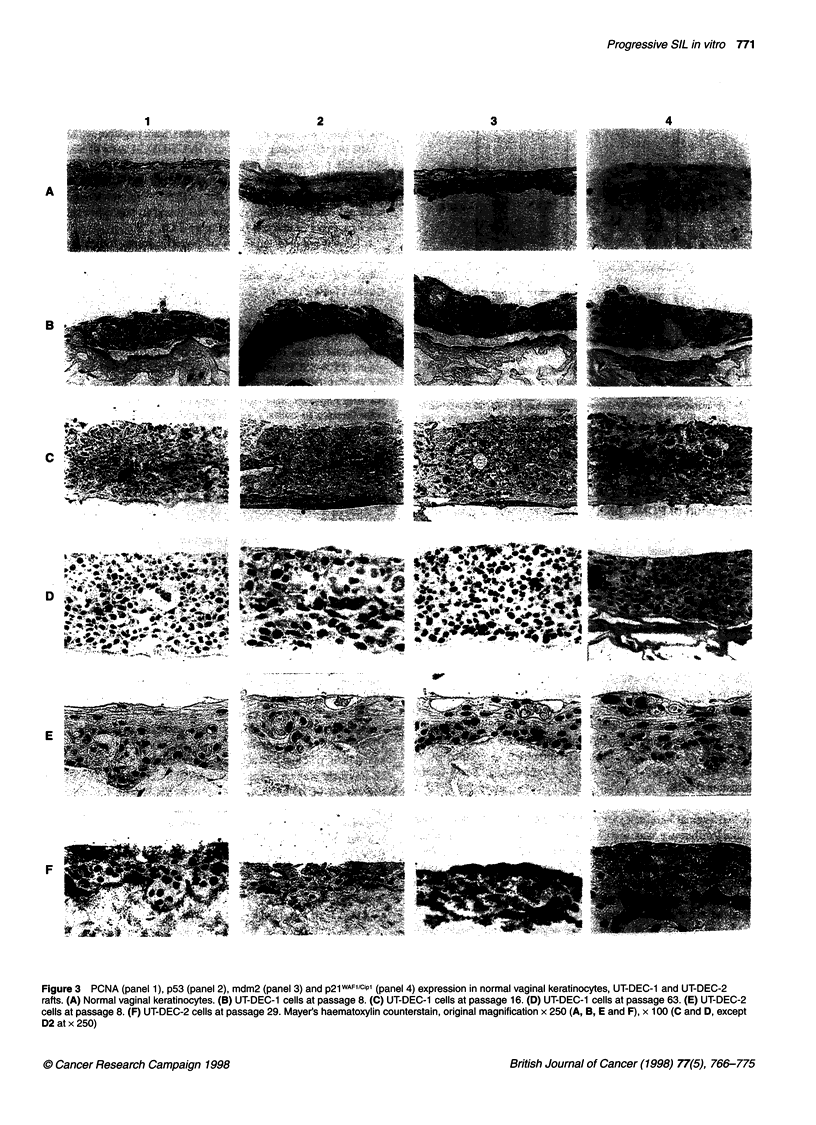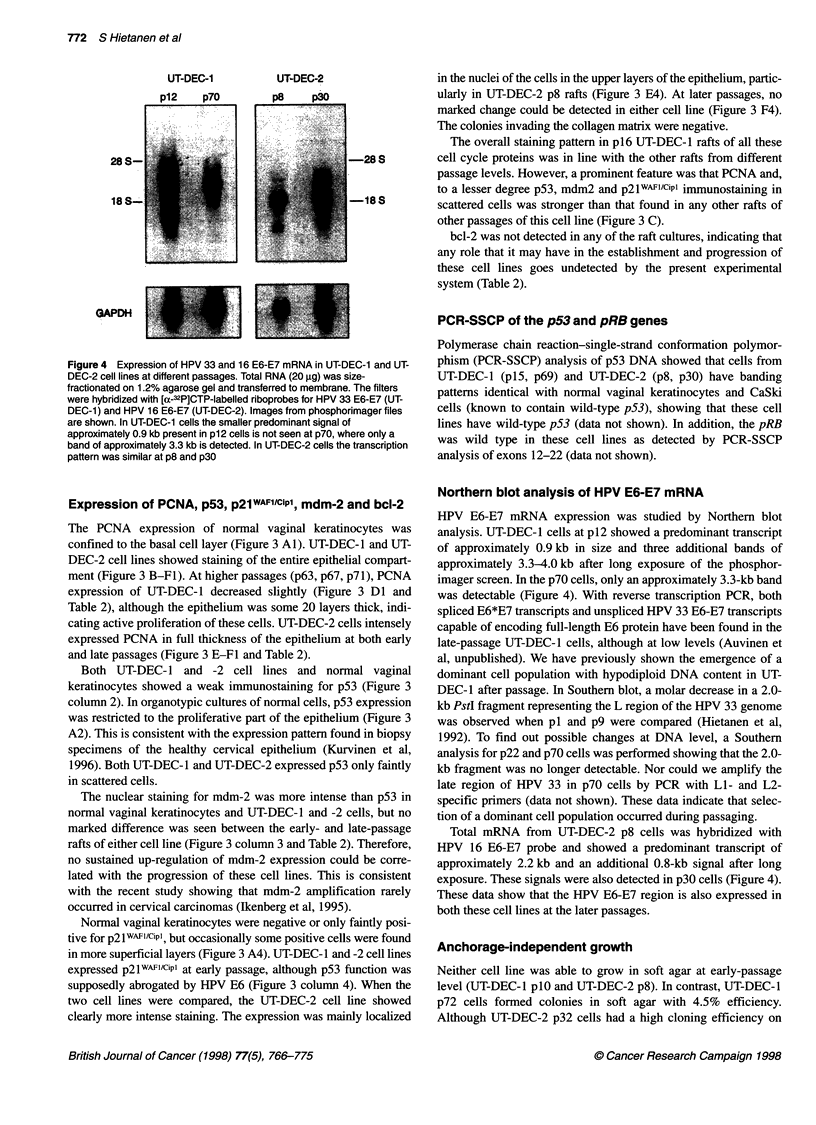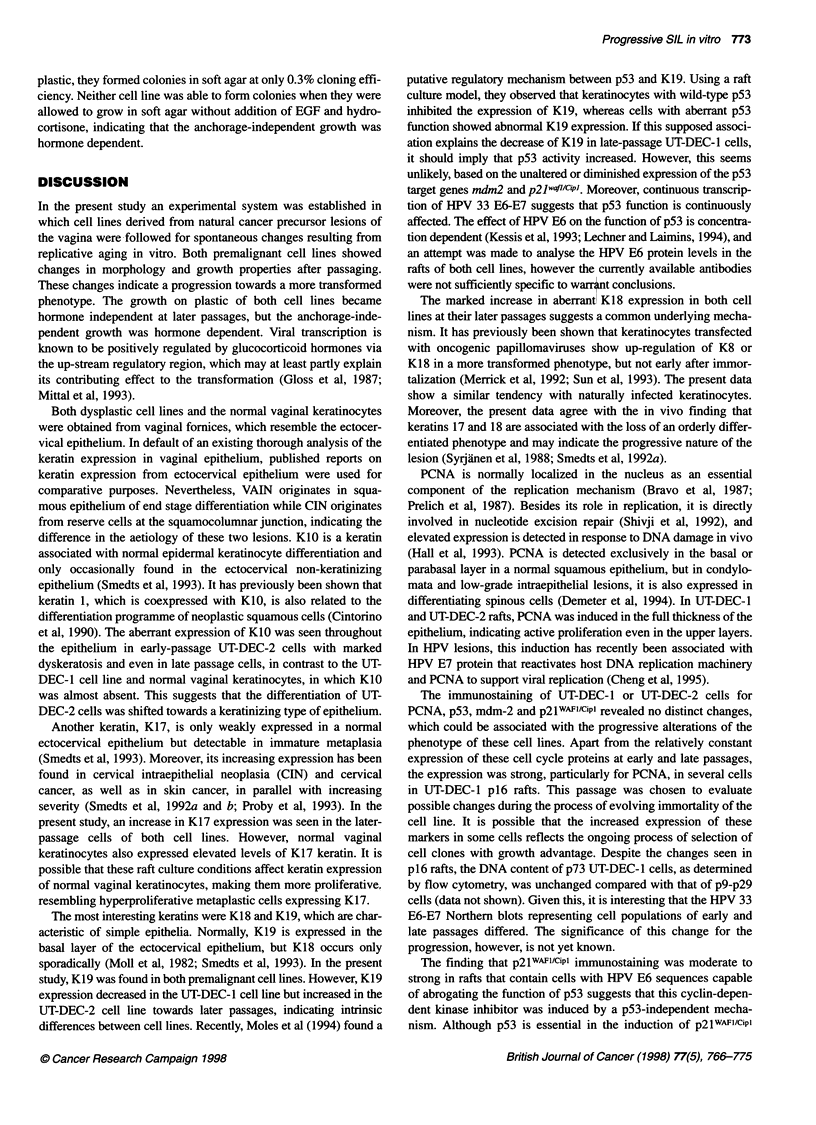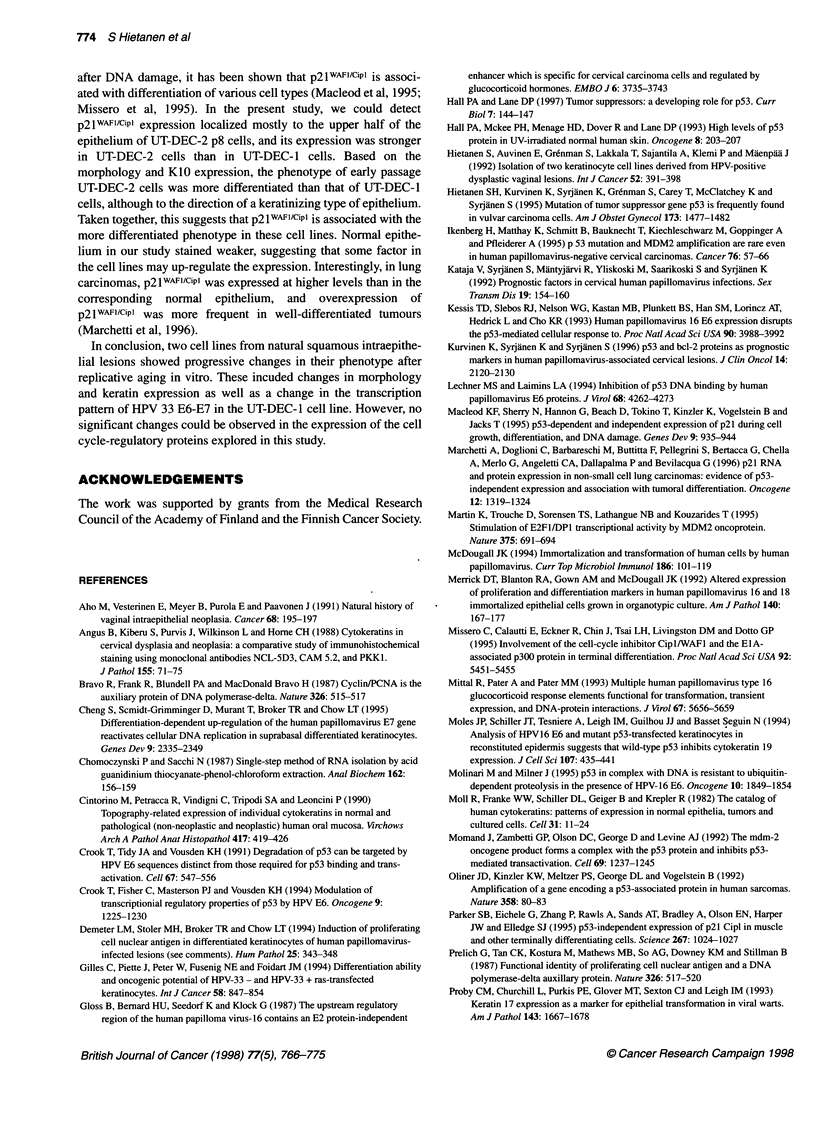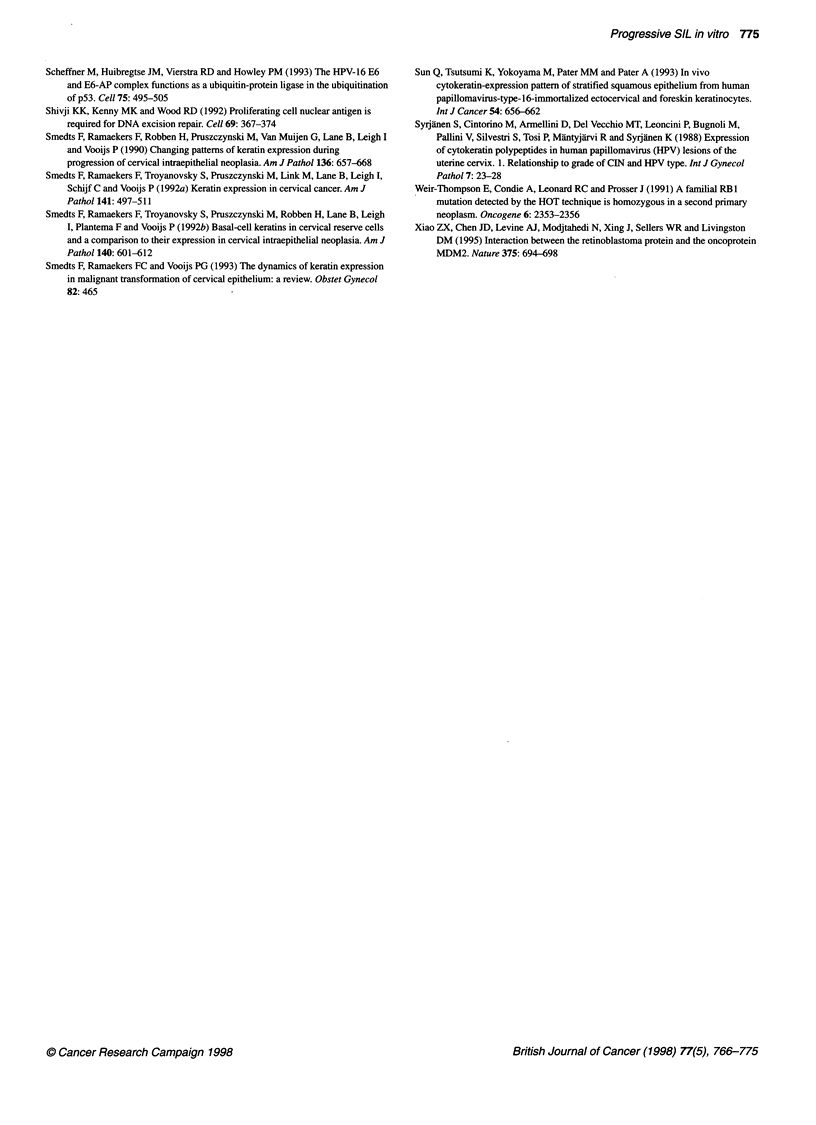# Characterization of keratin and cell cycle protein expression in cell lines from squamous intraepithelial lesions progressing towards a malignant phenotype.

**DOI:** 10.1038/bjc.1998.125

**Published:** 1998-03

**Authors:** S. Hietanen, K. SyrjÃ¤nen, S. SyrjÃ¤nen

**Affiliations:** Department of Obstetrics and Gynecology, Turku University Hospital, Finland.

## Abstract

**Images:**


*British Journal of Cancer* (1998) **77**(5), 766–775
© 1998 Cancer Research Campaign

### Characterization of keratin and cell cycle protein expression in cell lines from squamous intraepithelial lesions progressing towards a malignant phenotype

**S Hietanen[1,2], K Syrjänen[3] and S Syrjänen[3,4]**

[1]Department of Obstetrics and Gynecology, Turku University Hospital, Kiinamyllynkatu 2-4, 20520 Turku, Finland; [2]Medicity Research Laboratory, Turku University, Tykistökatu 6, 20520 Turku, Finland; [3]Department of Pathology, Kuopio University, PL 1627, 70211 Kuopio, Finland; [4]Department of Oral Pathology, Institute of Dentistry, Turku University, Lemminkäisenkatu 2, 20520 Turku, Finland

**Summary** Two cell lines derived from vaginal intraepithelial neoplasias (VAINs) expressing human papillomavirus (HPV) 33 (VAIN I, UT-DEC-1) and 16 (VAIN II, UT-DEC-2) E6-E7 mRNA were studied in organotypic culture for their keratins and cell cycle regulatory proteins in relation to replicative aging. Early-passage UT-DEC-1 and UT-DEC-2 cells reproduced epithelial patterns consistent with VAIN. Cells from later passages resembled full-thickness intraepithelial neoplasia (UT-DEC-1) and microinvasive cancer (UT-DEC-2). The morphological changes were compatible with these cell lines' ability for anchorage-independent growth at later passages. Simple epithelial keratins were aberrantly expressed in both cell lines. K18 (absent in normal vaginal keratinocytes) and K17 expression increased in UT-DEC-1 and UT-DEC-2 cells at late passages. No marked differences in expression of p53 (wild type in both cell lines), mdm-2 or PCNA were detected in parallel with progression. The expression of p21$^{WAF1/Cip1}$ localized mostly to the upper half of the epithelium at early passage and was more intense in the HPV 16-positive UT-DEC-2 cell line expressing K10. In Northern blot analyses, the transcription pattern of the HPV 33 E6-E7 of the UT-DEC-1 cell line changed during later passages, whereas that of the HPV 16 E6-E7 of the UT-DEC-2 cell line remained unaltered. The present characterization of the phenotype of these cell lines derived from natural squamous intraepithelial lesions shows an association between simple epithelial-type keratin expression and progressive changes in growth and morphology, but fails to demonstrate consistent changes in the expression of cell cycle regulatory proteins studied in parallel with progression.

**Keywords:** human papillomavirus; multistep carcinogenesis; malignant progression; organotypic culture

Human papillomaviruses (HPV) are small DNA viruses infecting squamous epithelia at various anatomical sites, including the skin and the mucosal linings of the anogenital tract. A subset of mucosal HPVs, including HPV types 16, 18, 31 and 33 (and others), is frequently associated with various grades of squamous intraepithelial neoplasia. Without therapeutic intervention, such lesions are known to progress to higher grades and invasive cancer, thus representing the premalignant stages in squamous cell carcinogenesis (Aho et al, 1991; Kataja et al, 1992).

The emergence of frankly malignant cell clones from target keratinocytes is accompanied by a variety of biochemical and morphological changes characterized by altered differentiation and loss of normal growth control (McDougall, 1994). During normal differentiation, keratinocytes are characterized by the coordinated expression of a variety of intermediate filaments, notably keratins, important cytoskeletal constituents of all epithelial cells. Human keratins, a family of at least 20 different molecules, are distributed in a tissue-specific fashion (Moll et al, 1982). Their differentiation-specific expression patterns have made them invaluable as markers of cell differentiation. Some previous studies have suggested that the behaviour of cervical intraepithelial neoplasia (CIN) may be predicted, based on keratin expression patterns (Angus et al, 1988; Syrjänen et al, 1988; Smedts et al, 1990).

*Received 27 January 1997*
*Revised 20 May 1997*
*Accepted 10 July 1997*

*Correspondence to:* S Hietanen, Medicity Research Laboratory, Turku University, Tykistökatu 6, 20520 Turku, Finland

Current molecular studies have shown the importance of tumour suppressor genes in the normal regulation of cellular proliferation and differentiation. The *p53* gene encodes a 53-kDa phosphoprotein, which acts as a negative regulator of cell proliferation after DNA damage, arresting the cell cycle via several target genes involved in cell cycle control, such as *GADD45* or *p21* (also known as *WAF1, CIP1* or *SDI1*, reviewed by Hall and Lane, 1997). Although the cyclin-dependent kinase inhibitor p21$^{WAF1/Cip1}$ has been proposed as the mediator of p53-induced cell cycle arrest after DNA damage, it has been shown that several stimuli now appear to induce its expression, independent of p53 function. The up-regulation of p21 has also been implicated during normal tissue development and differentiation (Macleod et al, 1995; Missero et al, 1995; Parker et al, 1995).

Interaction with HPV E6 affects p53 activity and may cause its degradation via the ubiquitin-dependent proteolytic system (Scheffner et al, 1993). E6 proteins of both oncogenic and benign HPV types associate in vitro with p53, but only binding by the E6 proteins of oncogenic HPV types can target p53 for degradation (Crook et al, 1991). Alternatively, E6 can block the ability of p53 to up-regulate the transcription of target genes without promoting its degradation (Crook et al, 1994; Lechner and Laimins, 1994; Molinari and Milner, 1995).

mdm-2 is a negative regulator of p53 activity (Momand et al, 1992). The ratio of p53 and mdm-2 is important in the regulation of normal cell division, but amplified mdm-2 can be found in some

**Table 1** Antibodies used for immunohistochemistry

| Antibody | Company | No. | Dilution | Pretreatment | Positive control sections |
|---|---|---|---|---|---|
| Cytokeratin 10 | Dako | M7002 | 1:10 | No pretreatment | Skin |
| Cytokeratin 17 | Dako | M7046 | 1:75 | Microwave, 10 mM citric acid, pH 6.0, 5 min | Oral cancer |
| Cytokeratin 18 | Dako | M7012 | 1:75 | Microwave, 10 mM citric acid, pH 6.0, 5 min | Prostate |
| Cytokeratin 19 | Dako | M888 | 1:500 | Trypsin 10 min, room temperature | Breast tissue |
| Collagen IV | Dako | M785 | 1:150 | Pepsin, 30 min, 37°C | Skin |
| Vimentin | Dako | M7020 | 1:500 | Trypsin 10 min, room temperature | Myometrium |
| PCNA PC10 | Dako | M879 | 1:1000 | No pretreatment | Oral mucosa |
| p53 DO7 | NovoCastra | NCL-p53-207 | 1:100 | Microwave, 10 mM citric acid, pH 6.0, 5 min | Breast cancer |
| mdm-2 | NovoCastra | NCL-MDM2 | 1:75 | Microwave, 10 mM tri-sodium citrate, pH 6.0, 5 min | Soft-tissue sarcoma |
| p21$^{WAF1/CIP1}$ | NovoCastra | NCL-WAF-1 | 1:20 | Microwave: 10 mM tri-sodium citrate, pH 6.0, 5 min | UV-irritated skin |
| bcl-2 | Dako | M887 | 1:100 | Microwave, 10 mM citric acid, pH 6.0, 5 min | Lymphoma |

human malignancies (Oliner et al, 1992). mdm-2 was recently found to interact physically with the other major tumour suppressor retinoblastoma gene product pRb and to augment proliferation by inducing transcription factor E2F (Martin et al, 1995; Xiao et al, 1995).

We were interested in the characterization of the expression patterns of keratins and growth-control proteins in HPV-positive cell lines derived from natural squamous intraepithelial lesions (SILs) grown in a raft culture system. The study was performed to evaluate whether any of the markers used in this study could be used to demonstrate stepwise changes of the epithelium towards a malignant phenotype in vitro.

### MATERIALS AND METHODS

### Cell lines

The two cell lines used in this study were derived from biopsy specimens of an HPV 33-positive VAIN I lesion (UT-DEC-1) and an HPV 16-positive VAIN II lesion (UT-DEC-2). The establishment and characterization of these cell lines have been described previously (Hietanen et al, 1992). At the time of establishment, the cells were cultured as monolayers in Dulbecco's modified Eagle medium (DMEM) supplemented with 1% non-essential amino acids, 2 mM L-glutamine, 50 μg ml$^{-1}$ streptomycin, 100 U ml$^{-1}$ penicillin, 1 μM dexamethasone, 10 ng ml$^{-1}$ human recombinant epidermal growth factor (EGF) and 10% fetal calf serum. From passage 19 onwards, UT-DEC-1 cells showed independence of growth factor addition and were maintained in the medium without dexamethasone or EGF. UT-DEC-2 required EGF addition to support growth, but dexamethasone was not added after passage 20.

For the raft culture studies, cryopreserved cells were thawed from early- and late-passage levels. The cells were allowed to expand in a monolayer culture, one to three passages before raft culture with a split ratio of 1:2. At this step, the medium was changed to a low-calcium, serum-free keratinocyte medium, a modification of MCDB 153 medium containing 50 μg ml$^{-1}$ bovine pituitary extract and 5 ng ml$^{-1}$ EGF (Keratinocyte-SFM, Gibco, Paisley, UK) to prevent the cells from further selection by differentiation before raft culture. Late-passage UT-DEC-1 cells were expanded in DMEM with 10% fetal bovine serum. Passage levels of the cell lines were selected so that they represented cells from the time during the establishment of the cell line as well as cells after extensive cell division. The passage 16 of UT-DEC-1 cells was chosen because of the integration of HPV 33 DNA into this cell line and consequent chromosomal instability detected previously in p8 cells (Hietanen et al, 1992). To date, UT-DEC-1 and UT-DEC-2 cell lines have undergone 75 and 33 passages respectively.

Normal vaginal keratinocyte cultures were maintained in Keratinocyte-SFM, and vaginal fibroblasts were grown in DMEM supplemented with 1% non-essential amino acids, 2 mM L-glutamine, 50 μg ml$^{-1}$ streptomycin, 100 U ml$^{-1}$ penicillin and 10% FBS. These normal cells were always used in the raft culture experiments before their fourth passage.

### Preparation of reorganized collagen gel

Vitrogen 100 collagen (Celtrix Pharmaceuticals, Santa Clara, CA, USA) was mixed with 10 × DMEM and neutralized by 0.1 M sodium hydroxide to pH 7.4 ± 0.2. Fibroblasts were suspended in the collagen solution at a cell density of 3 × 10$^5$ cells per 0.7 ml. This suspension was plated in 16-mm tissue culture dishes. The collagen–fibroblast suspension was allowed to gel at 37°C for 1 h. Then, the dishes were filled with DMEM supplemented with 5 μg ml$^{-1}$ insulin (Sigma, St Louis, MO, USA), 10 ng ml$^{-1}$ EGF (Boehringer Mannheim, Germany), 0.5 μg ml$^{-1}$ hydrocortisone (Sigma) and ascorbic acid 50 μg ml$^{-1}$ (Sigma) and maintained at 37°C in an atmosphere of 5% carbon dioxide at 90% relative humidity. The medium was changed three times per week for 1 week.

### Epidermal raft culture

Epithelial cells (2–3 × 10$^5$ cells per well) were added to the surface of the fibroblast–collagen gels. At least three parallel raft cultures were started from the same cells. In addition, both early- and late-passage cells were analysed at least twice on the rafts. The medium was changed to Green's medium and was identical for all individual rafts. The medium consisted of DMEM-Ham F12 3:1, 10% FBS (Gibco, BRL), 4 mM glutamine, 5 μg ml$^{-1}$ insulin, 0.18 mM adenine (Sigma), 0.4 μg ml$^{-1}$ hydrocortisone, 0.1 nM choleratoxin (Sigma) and 5 ng ml$^{-1}$ EGF. After the cells had reached confluence (usually in 2–4 days), the cultures were lifted into the air–liquid interface using a stainless steel grid. The medium was changed every 2–3 days. The medium was also changed in all raft cultures on the day preceding harvesting.

The raft cultures were allowed to stratify for 10 days and then harvested. Each raft was divided into two parts. One part was fixed in buffered 10% formalin for 24 h and the other snap-frozen and stored at –70°C for any future use. The formalin-fixed material was embedded in paraffin and processed into 5-μm sections for haematoxylin–eosin staining and immunohistochemistry.

© Cancer Research Campaign 1998

**Figure 1** Morphology of UT-DEC-1 and UT-DEC-2 cell lines and normal vaginal keratinocytes in an organotypic raft culture. **(A)** UT-DEC-1, passage 8. The polarity of the cells is slightly disturbed. No basal layer is seen. Some flattened cells are present in the superficial layer. **(B)** UT-DEC-1, passage 16: some apoptotic cells showing shrinking and chromatin condensation into perinuclear crescents (arrow). Basophilic chromatin fragments are seen sporadically, indicating the presence of apoptotic bodies (double arrow). Abnormal mitotic figures are also present (arrowhead). **(C)** UT-DEC-1, passage 63 cells from the UT-DEC-1 cell line. The morphology is consistent with a full-thickness intraepithelial neoplasia (VAIN III). **(D)** UT-DEC-2 cell line, passage 8. A cornified layer is present with marked dyskeratosis. **(E)** UT-DEC-2, passage 29: colonies of cells invading the collagen matrix are seen, sometimes without connection to the overlying epithelium. **(F)** Normal vaginal keratinocytes (H&E, original magnification × 250)

### Immunohistochemistry

The expression of a panel of keratins, collagen IV, vimentin, p53, p21$^{WAF1/CIP1}$, PCNA, mdm-2 and bcl-2 proteins was analysed in all raft cultures. Table 1 shows the characterization and dilutions of the monoclonal antibodies used for the immunohistochemical stainings.

Formalin-fixed, paraffin-embedded sections placed on organosilane-coated slides were deparaffinized in xylene and rehydrated with graded alcohol. Endogenous peroxidase activity was blocked using 5% hydrogen peroxide for 5 min. The sections were first incubated with 1.5% normal goat serum (Vector Laboratories, Burlingame, CA, USA) for 15 min (to reduce the non-specific antibody binding), followed by incubation with the primary antibody (in blocking serum) overnight at +4°C. At the next step, the sections were incubated with the secondary biotinylated antibody (anti-mouse IgG, Vector Laboratories) for 30 min, followed by incubation with an avidin–biotin–peroxidase complex (Vectastain Elite ABC Kit, Vector Laboratories) for 30 min. The immunoperoxidase reaction was developed using 3,3′-diaminobenzidine (DAB; Sigma) for 5 min. Finally, the sections were counterstained with Maeyer's haematoxylin, dehydrated and mounted with permount.

Positive and negative control sections were included in all stainings (Table 1). Sections treated with the blocking serum without primary antibodies served as negative controls. The intensity of immunoreactivity was graded as negative (–), low (+), moderate (++), strong (+++) or intense (++++). In addition, the distribution of positive epithelial cells (scattered or even distribution) was recorded separately.

### PCR-SSCP

The PCR conditions for the *p53* gene analysis and separation of the PCR products have been described in more detail previously (Hietanen et al, 1995). The analyses focused on exons 5–9 where most *p53* mutations in human cancers have been found. The primers for amplification of *pRB* were chosen as previously described and covered exons 12–22 (Weir-Thompson et al, 1991). The DNA of UT-DEC-1 cells from passages 15 and 69 and UT-DEC-2 cells from passages 8 and 30 were included in these analyses. DNA from normal vaginal keratinocytes were used as controls for the wild-type *p53* and *pRB*.

### RNA isolation and Northern blot analysis

Total RNA was isolated with Trizol (Life Technologies, Gaithersburg, MD, USA) from UT-DEC-1 and UT-DEC-2 grown in monolayer cultures according to the manufacturer's protocol based on the single-step method by Chomczynski and Sacchi (1987). RNA samples were treated with RNAase-free DNAase (Promega, Madison, WI, USA). Then, 20 µg of the RNA were fractionated in 1.2% agarose gel and transferred to Hybond N+ membrane (Amersham) by vacuum transfer (VacuGene XL; LKB, Bromma, Sweden) and immobilized by heating at 80°C. Filters were hybridized to $^{32}$P-labelled RNA probes transcribed in vitro. The HPV 16 E6-E7 probe (kindly provided by Dr M Dürst, German Cancer Research Center, Heidelberg, Germany) spans from the up-stream regulatory region to the entire E6-E7 ORFs of HPV 16. The cDNA for HPV 33 E6-E7 probe was synthesized from total cellular RNA from p72 UT-DEC-1 cells with primers spanning the HPV 33 E6-E7 region. A phosphorimager screen (BioRad, Hercules, CA, USA) was exposed for 20 h, whereafter specific mRNA hybridizations were detected (Molecular analyst, BioRad). After exposure, the probe was stripped from the membrane, and hybridization was then performed with a GAPDH probe labelled with random priming.

© Cancer Research Campaign 1998

### Anchorage-independent growth

UT-DEC-1 and UT-DEC-2 cells were diluted to $10^4$ cells per ml in medium containing 0.33% SeaPlague agar (FMC Bioproducts, Rockland, ME, USA), and 10 ml was plated on 85-mM dishes over a layer of 0.5% bottom agar dissolved in the medium. The medium consisted of DMEM with 10% FBS supplemented with 5 ng $ml^{-1}$ EGF and 0.5 μg $ml^{-1}$ hydrocortisone. The medium was changed twice weekly. After 2 weeks, colonies greater than ten cells were counted microscopically.

### RESULTS

### Morphology

*Normal cells*

Normal vaginal epithelial cells grown on the raft culture reproduced the growth pattern of a normal squamous epithelium, although the epithelium remained thinner (Figure 1F). Epithelial cells were organized in a thin sheet of five to seven cell layers thick. There was some tendency towards formation of a basal cell layer, although not as regular as that normally found in vivo. There was also a layer of flattened superficial cells, evidencing terminal differentiation of normal vaginal keratinocytes in this raft culture system.

*UT-DEC-1*

At early passage (p8), this premalignant squamous cell line grown on the raft culture reproduced an epithelial pattern with some resemblance to that of a normal vaginal epithelium (Figure 1A). The epithelial sheet was slightly thicker, however, and the arrangement (and polarity) of the cells was more disturbed, consistent with intraepithelial neoplasia (i.e. the origin of the cell line). No evidence of a basal cell layer could be found, but some tendency could be seen towards formation of a superficial layer of flattened cells.

Cells from p16, apart from their deranged epithelial architecture, showed several morphological changes typical of apoptosis (Figure 1B): some cells showed chromatin condensation, sometimes with crescent formation at the border of the nuclear membrane, and cellular shrinking. Occasionally, dense spheres could also be detected in the extracellular space, typical of apoptotic bodies (Figure 1B). Several cells showing abnormal mitoses were detected (Figure 1B). These changes were possibly related to cell line 'crisis' before evolution of immortality.

Cells taken from a later passage (p63, p67 and p71) grew notably differently. Epithelial thickness was greatly increased (up to 20 layers). No polarity was detectable, and the morphology was consistent with a full-thickness intraepithelial neoplasia (VAIN III, Figure 1C), although the cells originated from a VAIN I lesion. There was no evidence of terminal differentiation as indicated by the lack of any superficial cell layer. Compared with the early cultures, the late-passage cells showed marked dedifferentiation.

*UT-DEC-2*

Cells taken at an early passage (p8) of this cell line produced an epithelial structure of some eight to ten layers thick. The arrangement and polarity of cells were disturbed, but a cornified layer was still present with evidence of keratinization. Keratinization was also detected in the intermediate layer, indicating dyskeratosis (Figure 1D). In some sections, smaller basal-type cells were found, whereas at other sites the epithelial cells showed a tendency to penetrate into the underlying fibroblast–collagen matrix.

Cells from a later passage (p29) also formed a thin epithelium, with even more pronounced abnormal morphological features (Figure 1E). The nuclei were markedly enlarged with a hyperchromatic staining pattern. The raft from this passage was characterized by a definite tendency of cells for penetration (as tiny buds) into the underlying matrix (Figure 2 E2 and E4). Although originally derived from a VAIN II lesion, this cell line showed striking in vitro morphological progression with features consistent with an early invasive cancer.

### Expression of keratins

In the rafts of normal vaginal keratinocytes, weak expression of keratin 10 was seen infrequently only in some superficial epithelial cells or was totally absent (Figure 2 A1). Immunostaining was absent for K18 and weak for K19 (Figure 2 A3 and A4). Cells strongly positive for keratin 17 were found in all epithelial layers (Figure 2 A2). Collagen IV expression was detected as a faint band underlying the basal cell layer, as a sign of these cells tending to produce a structure resembling the basement membrane. Collagen IV was not detected in any of the UT-DEC-1 or UT-DEC-2 rafts (Table 2).

In some p8 UT-DEC-1 rafts, few faintly K10-positive cells were detectable among the superficial cells, but the expression was mostly absent (Figure 2 B1). K17 expression was faint but detectable throughout the epithelium (Figure 2 B2). K18 was very weakly expressed in scattered cells but mostly absent (Figure 2 B3), whereas moderate to strong K19 staining was detected primarily in the basal cell layer (Figure 2 B4).

At later passages of the UT-DEC-1 cell line (p63, p67 and p71), K10 was absent (Figure 2 C1). Intense K17 expression was detected in most epithelial cells (Figure 2 C2). The cultures of these late passages showed increasing intensity of K18 staining, and positive cells were present in all layers (Figure 2 C3). K19 antibodies produced only weak signals in the entire epithelium (Figure 2 C4).

When the p8 UT-DEC-2 cells were allowed to react with the K10 (a marker of epidermal keratinocyte differentiation) antibody, moderate to strong staining was detected in the upper half of the epithelium, but several cells were positive even in the vicinity of the basal layer (Figure 2 D1). The expression of K10 in early-passage UT-DEC-2 rafts was clearly more intense than that in early-passage UT-DEC-1 rafts. Strong K17 expression was detected in all layers (Figure 2 D2). A very weak K18 staining was seen in a few scattered cells (Figure 2 D3). K19 was moderately expressed in all cell layers (Figure 2 D4).

In the raft cultures of p29 UT-DEC-2, staining for K10 was weak (Figure 2 E1). K17 was intensely expressed in all layers (Figure 2 E2). K18 expression at p29 was much more intense than that at p8 (Figure 2 E3). Clusters of cells invading the collagen matrix also showed intense K18 staining. Similarly, K19 expression was increased and detected in most epithelial layers as well as in the invasive borders within the matrix (Figure 2 E4).

Another intermediate filament, vimentin, which is a marker for mesenchymal cytoskeleton, has been shown to be expressed in some transformed HPV 33-positive cell lines that have been extensively passaged (Gilles et al, 1994). In the present study, only fibroblasts in the collagen matrix stained with this antibody, but not the epithelial UT-DEC-1 and -2 cells (Table 2). The vimentin staining was also performed on monolayer cultures of both cell lines with similar negative results.

© Cancer Research Campaign 1998

**Figure 2** Immunohistochemical detection of keratins in normal vaginal keratinocytes and in UT-DEC-1 and UT-DEC-2 cell lines grown in an organotypic culture. Keratin 10 (panel 1), keratin 17 (panel 2), keratin 18 (panel 3) and keratin 19 (panel 4). **(A)** Normal vaginal keratinocytes. **(B)** UT-DEC-1 cells at passage 8. **(C)** UT-DEC-1 cells at passage 67. **(D)** UT-DEC-2 cells at passage 8. **(E)** UT-DEC-2 cells at passage 29 (Mayer's haematoxylin counterstain, original magnification × 250)

**Table 2** Immunohistochemical detection of intermediate filaments and cell cycle-regulatory proteins in UT-DEC-1 (HPV 33 positive), UT-DEC-2 (HPV 16 positive) and normal vaginal keratinocytes in organotypic raft cultures

| Passage of the cells used in the raft culture | K 10 | K 17 | K 18 | K 19 | Vimentin | Collagen IV | PCNA | p53 | mdm2 | $p21^{WAF1/CIP1}$ | bcl-2 |
|---|---|---|---|---|---|---|---|---|---|---|---|
| UT DEC-1 | | | | | | | | | | | |
| p8 | +/– | ++ | + | +++ | – | – | +++ | + | ++ | + | – |
| p16 | – | ++ | + | +++ | – | – | +++ | ++ | ++ | + | – |
| p63 | +/– | +++ | +++ | +++ | – | – | ++ | + | ++ | + | – |
| p67 | – | ++++ | +++ | ++ | – | – | ++ | + | + | + | – |
| p71 | – | ++++ | +++ | + | – | – | ++ | + | + | + | – |
| UT DEC-2 | | | | | | | | | | | |
| p8 | ++ | +++ | + | ++ | – | – | ++ | + | +++ | ++ | – |
| p29 | + | ++++ | +++ | ++++ | – | – | +++ | + | ++ | ++ | – |
| Normal vaginal keratinocytes | +/– | +++ | – | + | – | + | ++ | + | ++ | +/– | – |

–, No staining; +, weak; ++, moderate; +++, strong; ++++, intense staining.

 © Cancer Research Campaign 1998

**Figure 3** PCNA (panel 1), p53 (panel 2), mdm2 (panel 3) and p21$^{WAF1/Cip1}$ (panel 4) expression in normal vaginal keratinocytes, UT-DEC-1 and UT-DEC-2 rafts. **(A)** Normal vaginal keratinocytes. **(B)** UT-DEC-1 cells at passage 8. **(C)** UT-DEC-1 cells at passage 16. **(D)** UT-DEC-1 cells at passage 63. **(E)** UT-DEC-2 cells at passage 8. **(F)** UT-DEC-2 cells at passage 29. Mayer's haematoxylin counterstain, original magnification × 250 (**A, B, E** and **F**), × 100 (**C** and **D**, except **D2** at × 250)

*© Cancer Research Campaign 1998*

**Figure 4** Expression of HPV 33 and 16 E6-E7 mRNA in UT-DEC-1 and UT-DEC-2 cell lines at different passages. Total RNA (20 μg) was size-fractionated on 1.2% agarose gel and transferred to membrane. The filters were hybridized with [α-$^{32}$P]CTP-labelled riboprobes for HPV 33 E6-E7 (UT-DEC-1) and HPV 16 E6-E7 (UT-DEC-2). Images from phosphorimager files are shown. In UT-DEC-1 cells the smaller predominant signal of approximately 0.9 kb present in p12 cells is not seen at p70, where only a band of approximately 3.3 kb is detected. In UT-DEC-2 cells the transcription pattern was similar at p8 and p30

### Expression of PCNA, p53, p21$^{WAF1/Cip1}$, mdm-2 and bcl-2

The PCNA expression of normal vaginal keratinocytes was confined to the basal cell layer (Figure 3 A1). UT-DEC-1 and UT-DEC-2 cell lines showed staining of the entire epithelial compartment (Figure 3 B–F1). At higher passages (p63, p67, p71), PCNA expression of UT-DEC-1 decreased slightly (Figure 3 D1 and Table 2), although the epithelium was some 20 layers thick, indicating active proliferation of these cells. UT-DEC-2 cells intensely expressed PCNA in full thickness of the epithelium at both early and late passages (Figure 3 E–F1 and Table 2).

Both UT-DEC-1 and -2 cell lines and normal vaginal keratinocytes showed a weak immunostaining for p53 (Figure 3 column 2). In organotypic cultures of normal cells, p53 expression was restricted to the proliferative part of the epithelium (Figure 3 A2). This is consistent with the expression pattern found in biopsy specimens of the healthy cervical epithelium (Kurvinen et al, 1996). Both UT-DEC-1 and UT-DEC-2 expressed p53 only faintly in scattered cells.

The nuclear staining for mdm-2 was more intense than p53 in normal vaginal keratinocytes and UT-DEC-1 and -2 cells, but no marked difference was seen between the early- and late-passage rafts of either cell line (Figure 3 column 3 and Table 2). Therefore, no sustained up-regulation of mdm-2 expression could be correlated with the progression of these cell lines. This is consistent with the recent study showing that mdm-2 amplification rarely occurred in cervical carcinomas (Ikenberg et al, 1995).

Normal vaginal keratinocytes were negative or only faintly positive for p21$^{WAF1/Cip1}$, but occasionally some positive cells were found in more superficial layers (Figure 3 A4). UT-DEC-1 and -2 cell lines expressed p21$^{WAF1/Cip1}$ at early passage, although p53 function was supposedly abrogated by HPV E6 (Figure 3 column 4). When the two cell lines were compared, the UT-DEC-2 cell line showed clearly more intense staining. The expression was mainly localized in the nuclei of the cells in the upper layers of the epithelium, particularly in UT-DEC-2 p8 rafts (Figure 3 E4). At later passages, no marked change could be detected in either cell line (Figure 3 F4). The colonies invading the collagen matrix were negative.

The overall staining pattern in p16 UT-DEC-1 rafts of all these cell cycle proteins was in line with the other rafts from different passage levels. However, a prominent feature was that PCNA and, to a lesser degree p53, mdm2 and p21$^{WAF1/Cip1}$ immunostaining in scattered cells was stronger than that found in any other rafts of other passages of this cell line (Figure 3 C).

bcl-2 was not detected in any of the raft cultures, indicating that any role that it may have in the establishment and progression of these cell lines goes undetected by the present experimental system (Table 2).

### PCR-SSCP of the *p53* and *pRB* genes

Polymerase chain reaction–single-strand conformation polymorphism (PCR-SSCP) analysis of p53 DNA showed that cells from UT-DEC-1 (p15, p69) and UT-DEC-2 (p8, p30) have banding patterns identical with normal vaginal keratinocytes and CaSki cells (known to contain wild-type *p53*), showing that these cell lines have wild-type *p53* (data not shown). In addition, the *pRB* was wild type in these cell lines as detected by PCR-SSCP analysis of exons 12–22 (data not shown).

### Northern blot analysis of HPV E6-E7 mRNA

HPV E6-E7 mRNA expression was studied by Northern blot analysis. UT-DEC-1 cells at p12 showed a predominant transcript of approximately 0.9 kb in size and three additional bands of approximately 3.3–4.0 kb after long exposure of the phosphorimager screen. In the p70 cells, only an approximately 3.3-kb band was detectable (Figure 4). With reverse transcription PCR, both spliced E6*E7 transcripts and unspliced HPV 33 E6-E7 transcripts capable of encoding full-length E6 protein have been found in the late-passage UT-DEC-1 cells, although at low levels (Auvinen et al, unpublished). We have previously shown the emergence of a dominant cell population with hypodiploid DNA content in UT-DEC-1 after passage. In Southern blot, a molar decrease in a 2.0-kb *PstI* fragment representing the L region of the HPV 33 genome was observed when p1 and p9 were compared (Hietanen et al, 1992). To find out possible changes at DNA level, a Southern analysis for p22 and p70 cells was performed showing that the 2.0-kb fragment was no longer detectable. Nor could we amplify the late region of HPV 33 in p70 cells by PCR with L1- and L2-specific primers (data not shown). These data indicate that selection of a dominant cell population occurred during passaging.

Total mRNA from UT-DEC-2 p8 cells was hybridized with HPV 16 E6-E7 probe and showed a predominant transcript of approximately 2.2 kb and an additional 0.8-kb signal after long exposure. These signals were also detected in p30 cells (Figure 4). These data show that the HPV E6-E7 region is also expressed in both these cell lines at the later passages.

### Anchorage-independent growth

Neither cell line was able to grow in soft agar at early-passage level (UT-DEC-1 p10 and UT-DEC-2 p8). In contrast, UT-DEC-1 p72 cells formed colonies in soft agar with 4.5% efficiency. Although UT-DEC-2 p32 cells had a high cloning efficiency on

© Cancer Research Campaign 1998

plastic, they formed colonies in soft agar at only 0.3% cloning efficiency. Neither cell line was able to form colonies when they were allowed to grow in soft agar without addition of EGF and hydrocortisone, indicating that the anchorage-independent growth was hormone dependent.

### DISCUSSION

In the present study an experimental system was established in which cell lines derived from natural cancer precursor lesions of the vagina were followed for spontaneous changes resulting from replicative aging in vitro. Both premalignant cell lines showed changes in morphology and growth properties after passaging. These changes indicate a progression towards a more transformed phenotype. The growth on plastic of both cell lines became hormone independent at later passages, but the anchorage-independent growth was hormone dependent. Viral transcription is known to be positively regulated by glucocorticoid hormones via the up-stream regulatory region, which may at least partly explain its contributing effect to the transformation (Gloss et al, 1987; Mittal et al, 1993).

Both dysplastic cell lines and the normal vaginal keratinocytes were obtained from vaginal fornices, which resemble the ectocervical epithelium. In default of an existing thorough analysis of the keratin expression in vaginal epithelium, published reports on keratin expression from ectocervical epithelium were used for comparative purposes. Nevertheless, VAIN originates in squamous epithelium of end stage differentiation while CIN originates from reserve cells at the squamocolumnar junction, indicating the difference in the aetiology of these two lesions. K10 is a keratin associated with normal epidermal keratinocyte differentiation and only occasionally found in the ectocervical non-keratinizing epithelium (Smedts et al, 1993). It has previously been shown that keratin 1, which is coexpressed with K10, is also related to the differentiation programme of neoplastic squamous cells (Cintorino et al, 1990). The aberrant expression of K10 was seen throughout the epithelium in early-passage UT-DEC-2 cells with marked dyskeratosis and even in late passage cells, in contrast to the UT-DEC-1 cell line and normal vaginal keratinocytes, in which K10 was almost absent. This suggests that the differentiation of UT-DEC-2 cells was shifted towards a keratinizing type of epithelium.

Another keratin, K17, is only weakly expressed in a normal ectocervical epithelium but detectable in immature metaplasia (Smedts et al, 1993). Moreover, its increasing expression has been found in cervical intraepithelial neoplasia (CIN) and cervical cancer, as well as in skin cancer, in parallel with increasing severity (Smedts et al, 1992*a* and *b*; Proby et al, 1993). In the present study, an increase in K17 expression was seen in the later-passage cells of both cell lines. However, normal vaginal keratinocytes also expressed elevated levels of K17 keratin. It is possible that these raft culture conditions affect keratin expression of normal vaginal keratinocytes, making them more proliferative, resembling hyperproliferative metaplastic cells expressing K17.

The most interesting keratins were K18 and K19, which are characteristic of simple epithelia. Normally, K19 is expressed in the basal layer of the ectocervical epithelium, but K18 occurs only sporadically (Moll et al, 1982; Smedts et al, 1993). In the present study, K19 was found in both premalignant cell lines. However, K19 expression decreased in the UT-DEC-1 cell line but increased in the UT-DEC-2 cell line towards later passages, indicating intrinsic differences between cell lines. Recently, Moles et al (1994) found a putative regulatory mechanism between p53 and K19. Using a raft culture model, they observed that keratinocytes with wild-type p53 inhibited the expression of K19, whereas cells with aberrant p53 function showed abnormal K19 expression. If this supposed association explains the decrease of K19 in late-passage UT-DEC-1 cells, it should imply that p53 activity increased. However, this seems unlikely, based on the unaltered or diminished expression of the p53 target genes *mdm2* and *p21*$^{waf1/Cip1}$. Moreover, continuous transcription of HPV 33 E6-E7 suggests that p53 function is continuously affected. The effect of HPV E6 on the function of p53 is concentration dependent (Kessis et al, 1993; Lechner and Laimins, 1994), and an attempt was made to analyse the HPV E6 protein levels in the rafts of both cell lines, however the currently available antibodies were not sufficiently specific to warrant conclusions.

The marked increase in aberrant K18 expression in both cell lines at their later passages suggests a common underlying mechanism. It has previously been shown that keratinocytes transfected with oncogenic papillomaviruses show up-regulation of K8 or K18 in a more transformed phenotype, but not early after immortalization (Merrick et al, 1992; Sun et al, 1993). The present data show a similar tendency with naturally infected keratinocytes. Moreover, the present data agree with the in vivo finding that keratins 17 and 18 are associated with the loss of an orderly differentiated phenotype and may indicate the progressive nature of the lesion (Syrjänen et al, 1988; Smedts et al, 1992*a*).

PCNA is normally localized in the nucleus as an essential component of the replication mechanism (Bravo et al, 1987; Prelich et al, 1987). Besides its role in replication, it is directly involved in nucleotide excision repair (Shivji et al, 1992), and elevated expression is detected in response to DNA damage in vivo (Hall et al, 1993). PCNA is detected exclusively in the basal or parabasal layer in a normal squamous epithelium, but in condylomata and low-grade intraepithelial lesions, it is also expressed in differentiating spinous cells (Demeter et al, 1994). In UT-DEC-1 and UT-DEC-2 rafts, PCNA was induced in the full thickness of the epithelium, indicating active proliferation even in the upper layers. In HPV lesions, this induction has recently been associated with HPV E7 protein that reactivates host DNA replication machinery and PCNA to support viral replication (Cheng et al, 1995).

The immunostaining of UT-DEC-1 or UT-DEC-2 cells for PCNA, p53, mdm-2 and p21$^{WAF1/Cip1}$ revealed no distinct changes, which could be associated with the progressive alterations of the phenotype of these cell lines. Apart from the relatively constant expression of these cell cycle proteins at early and late passages, the expression was strong, particularly for PCNA, in several cells in UT-DEC-1 p16 rafts. This passage was chosen to evaluate possible changes during the process of evolving immortality of the cell line. It is possible that the increased expression of these markers in some cells reflects the ongoing process of selection of cell clones with growth advantage. Despite the changes seen in p16 rafts, the DNA content of p73 UT-DEC-1 cells, as determined by flow cytometry, was unchanged compared with that of p9-p29 cells (data not shown). Given this, it is interesting that the HPV 33 E6-E7 Northern blots representing cell populations of early and late passages differed. The significance of this change for the progression, however, is not yet known.

The finding that p21$^{WAF1/Cip1}$ immunostaining was moderate to strong in rafts that contain cells with HPV E6 sequences capable of abrogating the function of p53 suggests that this cyclin-dependent kinase inhibitor was induced by a p53-independent mechanism. Although p53 is essential in the induction of p21$^{WAF1/Cip1}$

© Cancer Research Campaign 1998

after DNA damage, it has been shown that p21$^{WAF1/Cip1}$ is associated with differentiation of various cell types (Macleod et al, 1995; Missero et al, 1995). In the present study, we could detect p21$^{WAF1/Cip1}$ expression localized mostly to the upper half of the epithelium of UT-DEC-2 p8 cells, and its expression was stronger in UT-DEC-2 cells than in UT-DEC-1 cells. Based on the morphology and K10 expression, the phenotype of early passage UT-DEC-2 cells was more differentiated than that of UT-DEC-1 cells, although to the direction of a keratinizing type of epithelium. Taken together, this suggests that p21$^{WAF1/Cip1}$ is associated with the more differentiated phenotype in these cell lines. Normal epithelium in our study stained weaker, suggesting that some factor in the cell lines may up-regulate the expression. Interestingly, in lung carcinomas, p21$^{WAF1/Cip1}$ was expressed at higher levels than in the corresponding normal epithelium, and overexpression of p21$^{WAF1/Cip1}$ was more frequent in well-differentiated tumours (Marchetti et al, 1996).

In conclusion, two cell lines from natural squamous intraepithelial lesions showed progressive changes in their phenotype after replicative aging in vitro. These incuded changes in morphology and keratin expression as well as a change in the transcription pattern of HPV 33 E6-E7 in the UT-DEC-1 cell line. However, no significant changes could be observed in the expression of the cell cycle-regulatory proteins explored in this study.

### ACKNOWLEDGEMENTS

The work was supported by grants from the Medical Research Council of the Academy of Finland and the Finnish Cancer Society.

### REFERENCES

Aho M, Vesterinen E, Meyer B, Purola E and Paavonen J (1991) Natural history of vaginal intraepithelial neoplasia. *Cancer* **68**: 195–197

Angus B, Kiberu S, Purvis J, Wilkinson L and Horne CH (1988) Cytokeratins in cervical dysplasia and neoplasia: a comparative study of immunohistochemical staining using monoclonal antibodies NCL-5D3, CAM 5.2, and PKK1. *J Pathol* **155**: 71–75

Bravo R, Frank R, Blundell PA and MacDonald Bravo H (1987) Cyclin/PCNA is the auxiliary protein of DNA polymerase-delta. *Nature* **326**: 515–517

Cheng S, Scmidt-Grimminger D, Murant T, Broker TR and Chow LT (1995) Differentiation-dependent up-regulation of the human papillomavirus E7 gene reactivates cellular DNA replication in suprabasal differentiated keratinocytes. *Genes Dev* **9**: 2335–2349

Chomoczynski P and Sacchi N (1987) Single-step method of RNA isolation by acid guanidinium thiocyanate-phenol-chloroform extraction. *Anal Biochem* **162**: 156–159

Cintorino M, Petracca R, Vindigni C, Tripodi SA and Leoncini P (1990) Topography-related expression of individual cytokeratins in normal and pathological (non-neoplastic and neoplastic) human oral mucosa. *Virchows Arch A Pathol Anat Histopathol* **417**: 419–426

Crook T, Tidy JA and Vousden KH (1991) Degradation of p53 can be targeted by HPV E6 sequences distinct from those required for p53 binding and trans-activation. *Cell* **67**: 547–556

Crook T, Fisher C, Masterson PJ and Vousden KH (1994) Modulation of transcriptionial regulatory properties of p53 by HPV E6. *Oncogene* **9**: 1225–1230

Demeter LM, Stoler MH, Broker TR and Chow LT (1994) Induction of proliferating cell nuclear antigen in differentiated keratinocytes of human papillomavirus-infected lesions (see comments). *Hum Pathol* **25**: 343–348

Gilles C, Piette J, Peter W, Fusenig NE and Foidart JM (1994) Differentiation ability and oncogenic potential of HPV-33 – and HPV-33 + ras-transfected keratinocytes. *Int J Cancer* **58**: 847–854

Gloss B, Bernard HU, Seedorf K and Klock G (1987) The upstream regulatory region of the human papilloma virus-16 contains an E2 protein-independent enhancer which is specific for cervical carcinoma cells and regulated by glucocorticoid hormones. *EMBO J* **6**: 3735–3743

Hall PA and Lane DP (1997) Tumor suppressors: a developing role for p53. *Curr Biol* **7**: 144–147

Hall PA, Mckee PH, Menage HD, Dover R and Lane DP (1993) High levels of p53 protein in UV-irradiated normal human skin. *Oncogene* **8**: 203–207

Hietanen S, Auvinen E, Grénman S, Lakkala T, Sajantila A, Klemi P and Mäenpää J (1992) Isolation of two keratinocyte cell lines derived from HPV-positive dysplastic vaginal lesions. *Int J Cancer* **52**: 391–398

Hietanen SH, Kurvinen K, Syrjänen K, Grénman S, Carey T, McClatchey K and Syrjänen S (1995) Mutation of tumor suppressor gene p53 is frequently found in vulvar carcinoma cells. *Am J Obstet Gynecol* **173**: 1477–1482

Ikenberg H, Matthay K, Schmitt B, Bauknecht T, Kiechleschwarz M, Goppinger A and Pfleiderer A (1995) p 53 mutation and MDM2 amplification are rare even in human papillomavirus-negative cervical carcinomas. *Cancer* **76**: 57–66

Kataja V, Syrjänen S, Mäntyjärvi R, Yliskoski M, Saarikoski S and Syrjänen K (1992) Prognostic factors in cervical human papillomavirus infections. *Sex Transm Dis* **19**: 154–160

Kessis TD, Slebos RJ, Nelson WG, Kastan MB, Plunkett BS, Han SM, Lorincz AT, Hedrick L and Cho KR (1993) Human papillomavirus 16 E6 expression disrupts the p53-mediated cellular response to. *Proc Natl Acad Sci USA* **90**: 3988–3992

Kurvinen K, Syrjänen K and Syrjänen S (1996) p53 and bcl-2 proteins as prognostic markers in human papillomavirus-associated cervical lesions. *J Clin Oncol* **14**: 2120–2130

Lechner MS and Laimins LA (1994) Inhibition of p53 DNA binding by human papillomavirus E6 proteins. *J Virol* **68**: 4262–4273

Macleod KF, Sherry N, Hannon G, Beach D, Tokino T, Kinzler K, Vogelstein B and Jacks T (1995) p53-dependent and independent expression of p21 during cell growth, differentiation, and DNA damage. *Genes Dev* **9**: 935–944

Marchetti A, Doglioni C, Barbareschi M, Buttitta F, Pellegrini S, Bertacca G, Chella A, Merlo G, Angeletti CA, Dallapalma P and Bevilacqua G (1996) p21 RNA and protein expression in non-small cell lung carcinomas: evidence of p53-independent expression and association with tumoral differentiation. *Oncogene* **12**: 1319–1324

Martin K, Trouche D, Sorensen TS, Lathangue NB and Kouzarides T (1995) Stimulation of E2F1/DP1 transcriptional activity by MDM2 oncoprotein. *Nature* **375**: 691–694

McDougall JK (1994) Immortalization and transformation of human cells by human papillomavirus. *Curr Top Microbiol Immunol* **186**: 101–119

Merrick DT, Blanton RA, Gown AM and McDougall JK (1992) Altered expression of proliferation and differentiation markers in human papillomavirus 16 and 18 immortalized epithelial cells grown in organotypic culture. *Am J Pathol* **140**: 167–177

Missero C, Calautti E, Eckner R, Chin J, Tsai LH, Livingston DM and Dotto GP (1995) Involvement of the cell-cycle inhibitor Cip1/WAF1 and the E1A-associated p300 protein in terminal differentiation. *Proc Natl Acad Sci USA* **92**: 5451–5455

Mittal R, Pater A and Pater MM (1993) Multiple human papillomavirus type 16 glucocorticoid response elements functional for transformation, transient expression, and DNA-protein interactions. *J Virol* **67**: 5656–5659

Moles JP, Schiller JT, Tesniere A, Leigh IM, Guilhou JJ and Basset Seguin N (1994) Analysis of HPV16 E6 and mutant p53-transfected keratinocytes in reconstituted epidermis suggests that wild-type p53 inhibits cytokeratin 19 expression. *J Cell Sci* **107**: 435–441

Molinari M and Milner J (1995) p53 in complex with DNA is resistant to ubiquitin-dependent proteolysis in the presence of HPV-16 E6. *Oncogene* **10**: 1849–1854

Moll R, Franke WW, Schiller DL, Geiger B and Krepler R (1982) The catalog of human cytokeratins: patterns of expression in normal epithelia, tumors and cultured cells. *Cell* **31**: 11–24

Momand J, Zambetti GP, Olson DC, George D and Levine AJ (1992) The mdm-2 oncogene product forms a complex with the p53 protein and inhibits p53-mediated transactivation. *Cell* **69**: 1237–1245

Oliner JD, Kinzler KW, Meltzer PS, George DL and Vogelstein B (1992) Amplification of a gene encoding a p53-associated protein in human sarcomas. *Nature* **358**: 80–83

Parker SB, Eichele G, Zhang P, Rawls A, Sands AT, Bradley A, Olson EN, Harper JW and Elledge SJ (1995) p53-independent expression of p21 Cipl in muscle and other terminally differentiating cells. *Science* **267**: 1024–1027

Prelich G, Tan CK, Kostura M, Mathews MB, So AG, Downey KM and Stillman B (1987) Functional identity of proliferating cell nuclear antigen and a DNA polymerase-delta auxillary protein. *Nature* **326**: 517–520

Proby CM, Churchill L, Purkis PE, Glover MT, Sexton CJ and Leigh IM (1993) Keratin 17 expression as a marker for epithelial transformation in viral warts. *Am J Pathol* **143**: 1667–1678

© Cancer Research Campaign 1998

Scheffner M, Huibregtse JM, Vierstra RD and Howley PM (1993) The HPV-16 E6 and E6-AP complex functions as a ubiquitin-protein ligase in the ubiquitination of p53. *Cell* **75**: 495–505

Shivji KK, Kenny MK and Wood RD (1992) Proliferating cell nuclear antigen is required for DNA excision repair. *Cell* **69**: 367–374

Smedts F, Ramaekers F, Robben H, Pruszczynski M, Van Muijen G, Lane B, Leigh I and Vooijs P (1990) Changing patterns of keratin expression during progression of cervical intraepithelial neoplasia. *Am J Pathol* **136**: 657–668

Smedts F, Ramaekers F, Troyanovsky S, Pruszczynski M, Link M, Lane B, Leigh I, Schijf C and Vooijs P (1992*a*) Keratin expression in cervical cancer. *Am J Pathol* **141**: 497–511

Smedts F, Ramaekers F, Troyanovsky S, Pruszczynski M, Robben H, Lane B, Leigh I, Plantema F and Vooijs P (1992*b*) Basal-cell keratins in cervical reserve cells and a comparison to their expression in cervical intraepithelial neoplasia. *Am J Pathol* **140**: 601–612

Smedts F, Ramaekers FC and Vooijs PG (1993) The dynamics of keratin expression in malignant transformation of cervical epithelium: a review. *Obstet Gynecol* **82**: 465

Sun Q, Tsutsumi K, Yokoyama M, Pater MM and Pater A (1993) In vivo cytokeratin-expression pattern of stratified squamous epithelium from human papillomavirus-type-16-immortalized ectocervical and foreskin keratinocytes. *Int J Cancer* **54**: 656–662

Syrjänen S, Cintorino M, Armellini D, Del Vecchio MT, Leoncini P, Bugnoli M, Pallini V, Silvestri S, Tosi P, Mäntyjärvi R and Syrjänen K (1988) Expression of cytokeratin polypeptides in human papillomavirus (HPV) lesions of the uterine cervix. 1. Relationship to grade of CIN and HPV type. *Int J Gynecol Pathol* **7**: 23–28

Weir-Thompson E, Condie A, Leonard RC and Prosser J (1991) A familial RB1 mutation detected by the HOT technique is homozygous in a second primary neoplasm. *Oncogene* **6**: 2353–2356

Xiao ZX, Chen JD, Levine AJ, Modjtahedi N, Xing J, Sellers WR and Livingston DM (1995) Interaction between the retinoblastoma protein and the oncoprotein MDM2. *Nature* **375**: 694–698

© Cancer Research Campaign 1998